# Measuring decision quality: psychometric evaluation of a new instrument for breast cancer chemotherapy

**DOI:** 10.1186/1472-6947-14-73

**Published:** 2014-08-20

**Authors:** Clara N Lee, Matthew H Wetschler, Yuchiao Chang, Jeffrey K Belkora, Beverly Moy, Ann Partridge, Karen R Sepucha

**Affiliations:** 1 Department of Surgery, Lineberger Comprehensive Cancer Center, Sheps Center for Health Services Research, University of North Carolina, UNC Plastic Surgery CB 7195, 27599-7195 Chapel Hill, NC, USA; 2 Department of Emergency Medicine, Stanford University, Palo Alto, USA; 3 General Medicine Division, Massachusetts General Hospital, Harvard Medical School, Boston, USA; 4 Institute for Health Policy Studies, University of California, San Francisco, CA, USA; 5 Massachusetts General Hospital Cancer Center, Harvard Medical School, Boston, USA; 6 Dana-Farber Cancer Institute, Brigham and Women’s Hospital, Harvard Medical School, Boston, USA; 7 Health Decision Sciences Center, Massachusetts General Hospital, Harvard Medical School, Boston, USA

**Keywords:** Decision, Regret, Breast cancer, Shared decision making, Quality of care, Decision quality, Chemotherapy, Adjuvant therapy

## Abstract

**Background:**

Women diagnosed with early stage (I or II) breast cancer face a highly challenging decision – whether or not to undergo adjuvant chemotherapy. We developed a decision quality instrument for chemotherapy for early stage breast cancer and sought to evaluate its performance.

**Methods:**

Cross-sectional, mailed survey of recent breast cancer survivors, providers, and healthy controls and a retest survey of survivors. The decision quality instrument includes questions on knowledge and personal goals. It results in a knowledge score and concordance score, which reflects the percentage of patients who received treatments that match their goals. Hypotheses related to acceptability, feasibility, validity, and reliability of the survey instrument were examined.

**Results:**

Responses were received from 352 patients, 89 providers and 35 healthy controls. The decision quality instrument was feasible to implement with few missing data. The knowledge scores had good retest reliability (intraclass correlation coefficient (ICC) =0.75). Knowledge scores discriminated between providers and patients (mean difference 31.1%, 95% CI 26.9, 35.3) and between patients and healthy controls (mean difference 11.2, 95% CI 5.4, 17.1). Most providers reported that the knowledge items covered essential content. Two of the five goal items had a ceiling effect, and one goal had low content validity. The goal items had moderate retest reliability (ICC’s 0.57 to 0.78). In the multivariable model of treatment, none of the patient goals was associated with receipt of chemotherapy. Age and hormone receptor status were the only variables independently associated with chemotherapy. Most patients (77.6%) had treatment concordant with that predicted by the model. Patients who had concordant treatment had similar levels of confidence and regret as those who did not.

**Conclusions:**

The Decision Quality Instrument is a reliable and valid measure of patient knowledge about chemotherapy, but its ability to measure concordance with patient goals is limited. In this sample, patient goals were not associated with treatment, and most patients reported they were not asked their preference, suggesting that goals were not adequately considered in decision making.

## Background

Women diagnosed with early stage (I or II) breast cancer face a highly challenging decision – whether or not to undergo adjuvant chemotherapy. Chemotherapy can provide gains in survival after breast cancer, but it also poses risks of serious toxicity. Because of this tradeoff between survival gains and toxicity risks, clinical guidelines leave the decision about chemotherapy open to the patient and provider, for most patients with stage I disease [1,2]. For Stage I patients, five year survival is quite high, and chemotherapy has a small absolute impact on 10-year survival [3-5]. Thus, the decision about chemotherapy for such patients is a “preference sensitive” one, for which medical evidence is necessary but not sufficient, and the right choice depends heavily on the patient’s personal preferences [6]. For patients with stage II disease, guidelines clearly recommend chemotherapy [1]. For those patients, the decision about chemotherapy should be an informed one, for which the patient fully understands the risks and benefits of the treatment.

Breast cancer patients report challenges and dissatisfaction in making decisions about their treatment [7], and some patients have significant gaps in knowledge or unrealistic expectations of the benefits of systemic therapy [8-12]. Patients tend to rely heavily on provider recommendations about chemotherapy [13], but providers do not always know or elicit patients’ preferences [14] and they may convey information in ways that patients do not understand [15]. Overall, the evidence suggests that early-stage patients may not be meeting acceptable standards for informed consent when making decisions with their providers about adjuvant chemotherapy.

Reducing the rate of uninformed or preference-insensitive chemotherapy decisions will require better measures of decision quality, due to a lack of reliable measures in these domains. A recent international consensus process has defined decision quality as *the extent to which patients are informed and receive treatments that match their goals*[6]. Valid measures of patient knowledge and preferences for breast cancer chemotherapy decisions, however, are lacking. Most studies of patient preferences have focused on the minimum survival gain that patients require to accept chemotherapy and have not examined the full set of goals and concerns that patients express, including risks of complications and side effects [8,16-19]. Studies of knowledge have relied on *ad hoc* measures [8].

To address the need for better assessment of quality in chemotherapy decisions, we developed a survey instrument to measure patient knowledge and preferences about chemotherapy for early stage breast cancer, and evaluated its psychometric properties in a field test in breast cancer survivors. The instrument results in two scores, a knowledge score, indicating the extent to which patients are informed, and a concordance score, indicating the percentage of patients who received treatments that matched their goals. To compare the instrument’s performance in groups with different levels of knowledge, we also surveyed breast cancer providers and healthy volunteers. The purpose of the field test was to evaluate feasibility, validity, and reliability of a new instrument that has never been used with breast cancer patients. Given the stress and vulnerability of newly diagnosed patients, we decided to conduct the field test with patients who had recovered from treatment. This approach would provide sufficient data to determine whether the questions were performing as intended, identify problematic wording or other issues, and allow some freedom to include more items than might be reasonably asked of newly diagnosed patients.

## Methods

### Initial instrument development

The approach to measuring decision quality draws from normative and behavioral decision making theories and is based on the conceptual framework of shared decision making, as described by Mulley [20] and extended by Mulley and Sepucha [21]. Decision quality is defined as the extent to which patients are informed and receive treatments that match their goals [6]. Thus the two primary domains of the instruments are knowledge of key facts and incorporation of patient goals and concerns into the decision.

Development of the Decision Quality Instrument (BCS-DQI©) followed best practices of survey research methods [22-24]. We derived the initial set of facts and goals from a thorough review of the clinical literature and from focus groups of breast cancer patients. The candidate facts and goals were then rated by convenience samples of patients (n = 17) and a multidisciplinary group of providers (n = 27) to evaluate accuracy, importance and completeness [25,26]. Experts in survey research drafted items to cover the key content and conducted cognitive interviews with patients (n = 6) in order to ensure comprehension and acceptability. Of the patients who participated in cognitive interviews, two had Stage I disease, 3 had Stage II, and one had unknown stage. Time since diagnosis ranged from 6 months to 5 years.

### Study design

The instrument was tested in a cross-sectional survey of breast cancer patients and providers at four National Cancer Institute-designated cancer centers in 2008, along with healthy volunteers from one of the sites. The four cancer centers were academically affiliated and located in urban areas in the Northeast, Southeast, and West Coast of the United States. The study was approved by the Institutional Review Boards at the Dana Farber Cancer Institute, Massachusetts General Hospital, University of California San Francisco, and University of North Carolina Chapel Hill.

### Samples and procedures

Eligible patients were adult women, diagnosed in 2005 or 2006 with early stage breast cancer, as reported in the cancer registry at each site. Exclusion criteria were a diagnosis prior to 2005, Stage 3 or higher, ductal carcinoma in situ only, bilateral breast cancer, receipt of neoadjuvant(before surgery) chemotherapy, and inability to read or speak English. The patients’ treating physicians approved contact.

Eligible patients received the study packet, including an informed consent form, in the mail. Respondents returned their signed informed consent form, signed authorization to access the medical record, and completed study questionnaire by mail. After two weeks, the study staff called those who did not opt out. After four weeks, non-respondents received a reminder mailing and follow up phone call. Respondents received a thank-you letter and small compensation (e.g., $8 book of stamps) for completing the survey. A subset of respondents across all sites received a second survey by mail approximately four weeks after responding, to assess test-retest reliability of the decision quality instrument.

A multidisciplinary group of breast cancer providers (medical oncologists, radiation oncologists, surgical oncologists, plastic surgeons, and general surgeons) was identified by the principal investigator at each site. Healthy volunteers who had no history of breast cancer and were not clinicians were recruited through employee email bulletins of Partners Healthcare in Massachusetts. Providers and healthy volunteer participants received the knowledge portion of the survey by mail. All potential participants received an email reminder after two weeks and a mailed reminder after four weeks. Providers received $50 cash or a gift card upon completion of the survey. Healthy volunteers received a book of stamps upon completion.

### Measures

We collected data on demographics and treatment history from each site’s cancer registry and the patient’s report on the survey. When patient self-report conflicted with the registry on a medical or treatment issue (e.g., stage of disease), we examined the medical record to resolve the issue. The survey included the following measures:

1. Breast Cancer Systemic Therapy Decision Quality Instrument (BCS-DQI ©, see Additional file 1). Items cover decisions about adjuvant chemotherapy and hormone therapy. Here we report on the chemotherapy items only. Findings regarding the hormone therapy items will be reported separately. The instrument has two primary domains:

a. Knowledge: 8 multiple choice and 4 open-ended items about recurrence, survival, treatment processes, and risks associated with chemotherapy.

b. Goals and concerns: 5 items rating on a scale from 0 (not at all important) to 10 (extremely important).

2. Decision process: 4 items about the nature of the interaction with health care providers including discussion of chemotherapy, pros and cons, and patients’ preferences. In addition, 1 item asked patients about who made the final decision about treatments (mainly the doctor, mainly me, both equally).

3. Patient’s preferred treatment: A single item asked, “Which treatment was your personal preference?” with 4 possible responses: chemotherapy only, hormone therapy only, both chemotherapy and hormone therapy, not have chemotherapy and not have hormone therapy*.*

4. Treatment received: A single item asking “Did you have chemotherapy?”

5. Informed: A single item asking “How informed did you feel about chemotherapy treatments?” with a 0 to 10 rating scale where 0 (not informed at all) and 10 (extremely well informed).

Confidence: A single item asking “How confident were you that the decision about chemotherapy was the right one?” with a 0 to 10 rating scale where 0 (not confident at all) and 10 (extremely confident). This item has been used in large national studies of decision making. Those studies found that patients reporting a stronger treatment preference and more involvement in decision making had higher confidence [27,28].

6. Regret: A single item asking “If you had to do it again, would you have the same treatments?” with a 5-point Likert scale. The response options were: definitely have the same, probably have the same, not sure, probably not have the same, and definitely not have the same. This item has been used in national studies of decision making that found higher preference concordance was associated with lower regret [29].

Healthy Volunteer survey: Healthy volunteers completed the knowledge questions and some demographic items.

Provider survey: Providers completed the knowledge questions, reported how important each item was (essential, important, not important), and rated how well the knowledge questions covered key content (extremely well, very well, somewhat well, not at all well). We did not ask providers to suggest additional knowledge items, since we had done that in prior stages of instrument development [26].

### Analysis

#### Sample evaluation

We began by evaluating demographics and response rates of the three samples (patient, provider, healthy volunteer).

#### Item retention and deletion

We evaluated each item in terms of problems with format, level of difficulty, redundancy, and floor and ceiling effects. A problem with format was defined as more than 5% of respondents having a problem, such as marking two answers when one was expected. A knowledge item was identified as possibly too difficult if fewer than 50% of providers responded correctly, or too easy if more than 80% of healthy volunteers answered correctly. An item was identified as possibly redundant if inter-item correlation was greater than 0.8. A steering committee composed of six experts in survey research, decision sciences, and breast cancer care (including two of the authors KS and CL) reviewed these analyses and recommended items for revision or deletion.

#### Knowledge score

A total knowledge score for each patient was calculated, using the items that were retained after the item retention and deletion process. The knowledge score was defined as the percentage of knowledge questions answered correctly. Missing responses to the knowledge items were considered incorrect, and any respondent who did not complete at least half of the knowledge items did not receive a knowledge score. An aggregate total knowledge score (0-100%) was computed for each sample.

#### Concordance score

We limited the concordance analysis to Stage I patients since chemotherapy has modest absolute impact on their year survival [3-5], and guidelines recommend considering (rather than definitely providing) chemotherapy, so their decisions are more preference-sensitive. The concordance analysis began by examining each goal to determine whether or not it distinguished between those who did and did not have chemotherapy. Then we included the goals, along with age, and hormone receptor status (defined as estrogen and progesterone receptor positive or not) in a multivariable logistic regression model, with receipt of chemotherapy as the dependent variable. To take into account correlation of observations from the same hospital, a Generalized Estimating Equations (GEE) approach was used with study site as a clustering variable. A patient who had predicted probability ≥0.5 and received chemotherapy, or who had predicted probability <0.5 and did not receive chemotherapy, was defined as having care that “matched” her goals. A summary concordance score (0-100%) was calculated by dividing the number of patients who matched by the total number of patients.

In addition to this concordance analysis in all Stage I patients, we performed an additional analysis in Stage I patients with hormone receptor-negative tumors, since guidelines provide stronger recommendations for chemotherapy in these patients [1].

#### Acceptability and feasibility

We examined acceptability using instrument completion time, response rates, and written comments. We only had an estimate of completion time because participants had reported completion time for the whole survey, which included scales other than the systemic therapy DQI. We evaluated feasibility using item non-response patterns. Any item with more than 5% missing responses was subject to revision.

#### Assessment of reliability

To examine retest reliability, we calculated the intra-class correlation coefficient for the total knowledge score and for the individual goals and concerns. The target was to exceed 0.7. Because the knowledge items were not based on a single underlying construct, we did not calculate Cronbach’s alpha or evaluate internal consistency.

#### Validity of knowledge score

For discriminant validity of the knowledge items, we tested the hypothesis that the provider total knowledge score would be higher than the patient score, and the patient score would be higher than the healthy volunteer score (using ANOVA with planned comparison). For content validity, we evaluated whether or not at least 70% of providers reported that the knowledge items covered key content.

#### Validity of concordance score

This analysis was limited to Stage I patients. For construct validity of the concordance model, we tested whether or not patients who stated a preference for chemotherapy had a higher model-predicted probability of chemotherapy than those who were unsure, using a two-sample *t*-test. We examined the hypothesis that concordance would be associated with less regret using a Chi-squared test. We also tested the hypothesis that concordance would be associated with more confidence in decisions using a two-sample *t*-test. For content validity of the goal items, we evaluated whether or not at least 20% of patients included each item in their top three most important goals.

## Results

### Sample characteristics

This study was part of a larger study of breast cancer treatment decisions, in which some patients received the survey on systemic therapy, and others received a survey on breast reconstruction. In the overall study, 456 out of 768 patients (59.4%) participated in the initial survey, and 125/137 (91.2%) in the retest survey. This paper includes 352 who completed the systemic therapy initial survey and 89 systemic therapy retest surveys. Many of those patients (237 of 352, 67.3%) had Stage I disease and were included in the concordance analyses. Among the Stage I patients, 35.4% received chemotherapy, alone or in combination with hormone therapy (Table 1). The rate of chemotherapy did not vary by site, for Stage I or Stage II patients. The mean length of time since diagnosis was 30.8 months (SD 9.8). The sample of 35 healthy volunteers was similar to the patient sample, in terms of race, education, marital status, and reported health status, but was younger on average (42 vs. 57 years, p <0.001) (Table 1). Most providers who received the survey completed it (89 of 116, 77%). The providers’ average age was 45 years (SD 9), most were female (65%), and they had been in practice an average of 15 years (SD 11). The provider sample was multidisciplinary, including medical oncologists (33%), nurses (29%), surgeons/surgical oncologists(17%), radiation oncologists (8%) and plastic surgeons (8%).

**Table 1:** Characteristics of patients and healthy volunteers

	**Patients**						**Healthy volunteers**	
	**Stage I**		**Stage II**		**Total**			
All	237	(67.3)	115	(32.7)	352	(100.0)	35	
Age mean (SD)	57	(11)	57	(12)	57	(11)	42.4	(11)
Race, n (%)								
White	199	(84.0)	92	(80.0)	291	(82.7)	26	(74.3)
Black	17	(7.2)	13	(11.3)	30	(8.5)	3	(8.6)
Hispanic	7	(3.0)	2	(1.7)	9	(2.6)	1	(2.9)
Asian, Hawaiian, Pacific Islander	7	(3.0)	4	(3.5)	11	(3.1)	3	(8.6)
Other/unknown	7	(3.0)	4	(3.5)	11	(3.1)	2	(5.7)
Education, n (%)								
HS or less	29	(12.2)	13	(11.3)	42	(11.9)	0	(0.0)
Some college	51	(21.5)	36	(31.3)	87	(24.7)	8	(22.9)
College or more	157	(66.2)	66	(57.4)	223	(63.4)	27	(77.1)
Hormone receptor status, n (%)								
Positive	198	(83.5)	96	(83.5)	294	(83.5)	na	
Negative	39	(16.5)	19	(16.5)	58	(16.5)	na	
Local treatment, n (%)								
Lumpectomy	172	(72.6)	80	(69.6)	252	(71.6)	na	
Mastectomy	65	(27.4)	35	(30.4)	100	(28.4)	na	
Systemic therapy, n (%)								
Chemotherapy only	33	(13.9)	21	(18.3)	54	(15.3)	na	
Hormone therapy only	124	(52.3)	22	(19.1)	146	(41.5)	na	
Both	51	(21.5)	65	(56.5)	116	(33.0)	na	
No systemic therapy	29	(12.2)	7	(6.1)	36	(10.2)	na	
Months since diagnosis								
Median (25th, 75th)	29	(23,38)	31	(22,38)	30	(23,38)	na	
Overall Health, n (%)								
Excellent/very good	159	(67.1)	70	(60.9)	229	(65.1)	32	(91.4)
Other	78	(32.9)	45	(39.1)	123	(34.9)	3	(8.6)

*Abbreviations*: *na* not applicable; *SD* standard deviation.

### Item retention and deletion

Two knowledge items were deleted for being too easy – “Can breast cancer come back after chemotherapy?” (correct answer “yes”) and “Mark whether or not some women have this side effect from chemotherapy: a. hair loss” (correct answer“yes”). We also recommended reducing the number of open-ended quantitative estimate questions (e.g., “If 100 women with breast cancer like yours had chemotherapy, about how many would be cancer free in ten years? ___ write in number of people”) from 4 to 2, because of feedback that patients had difficulty distinguishing among the four recurrence/survival scenarios, and because the questions did not add more information. We kept some of the open-ended questions because they were on critical topics, such as recurrence.

We recommended removal of the response option “I am not sure” because patients used it so frequently (frequency ranging from 11.4 to 49.4%) (Table 2), and it appeared to result in underestimation of patient knowledge.

**Table 2:** Selected knowledge items and responses (of 12 total items)

Question and possible responses (correct response in italics)*	**Number with this response, n (%)**					
	**Patients**		**Providers**		**Controls**	
N	352		89		35	
How much would waiting 4 weeks to make a decision about chemotherapy and hormone therapy affect chances of survival?						
A lot	36	(10.2)	0	(0)	6	(17)
Somewhat	54	(15.3)	5	(6)	14	(40)
*A little or not at all*	215	(61.1)	81	(91)	13	(37)
I am not sure	40	(11.4)	1	(1)	2	(6)
Missing	7	(2.0)	1	(1)	0	(0)
Without chemotherapy or hormone therapy, about how many women with early stage breast cancer will eventually die of breast cancer?						
More than half	52	(14.8)	5	(6)	14	(40)
About half	44	(12.5)	7	(8)	7	(20)
*Less than half*	105	(29.8)	70	(79)	10	(29)
I am not sure	144	(40.9)	3	(3)	4	(11)
Missing	7	(2.0)	4	(4)	0	(0)
Without chemotherapy or hormone therapy, about how many women with early stage breast cancer will be cancer free in ten years?						
*More than half*	77	(21.9)	48	(54)	4	(11)
About half	44	(12.5)	15	(17)	3	(9)
Less than half	76	(21.6)	18	(20)	21	(60)
I’m not sure	146	(41.5)	4	(4)	7	(20)
Missing	8	(2.3)	4	(4)	0	(0)
Out of 100 women who have chemotherapy, how many will have a serious side effect, such as another cancer or serious heart problem?						
*Fewer than 5*	70	(19.9)	59	(66)	5	(14)
5 to 10	63	(17.9)	15	(17)	12	(34)
11 to 20	23	(6.5)	4	(4)	7	(20)
More than 20	18	(5.1)	1	(1)	4	(11)
I am not sure	166	(47.2)	7	(8)	7	(20)
Missing	10	(2.8)	3	(3)	0	(0)
If 100 women with early breast cancer did not have chemotherapy or hormone therapy, how many would be cancer free in 10 years? (___ write in number)						
Answer less than 60	139	(39.5)	18	(20)	28	(80)
*Answer within 60–90 range*	79	(22.4)	31	(35)	1	(3)
Answer greater than 90	13	(3.7)	0	(0)	0	(0)
Not sure	115	(32.7)	15	(17)	6	(17)
Missing	6	(1.7)	25	(28)	0	(0)
Total knowledge score, mean (SD)	40	(21.0)	71	(17)	29	(16)

*Some questions have been shortened to fit this table.

Two of the goal items had a ceiling effect. For “Live as live as long as possible,” 284 of 352 patients (79.5%) rated the importance as 10 out of 10. For “Lower chance of recurrence”, 311 of 354 (88.4%) rated the importance as 10 out of 10. We kept these items for the concordance analyses because most patients included them in their top three issues. However, the steering committee recommended revision in future versions to improve their ability to discriminate between patients who had chemotherapy and those who did not.

At least 20% of patients selected each of the goals, except one, in their top three. One goal, “Avoid the costs of having additional treatments”, was deleted for lack of content validity -- only 5.4% of respondents selected it in their top three, and its selection did not vary by treatment. Patients who did not undergo chemotherapy had a slightly different frequency of selecting goals in their top three, compared to those who did undergo chemotherapy. For example, fewer patients who did not have chemotherapy selected “live as long as possible” (84.1 for those who did not have chemotherapy versus 92.4% for those who did, p = 0.021) and “lower the chance of having cancer come back” (85.7% vs. 98.2%, p = 0.001), in their top three goals.

### Acceptability and feasibility

The estimated mean completion time for the DQI (including the knowledge items, goals and decision process items) was 7.2 minutes (range 2.3, 22.9 minutes). The knowledge items had a mean of 2.5% missing responses (range 1.4 to 4.5%). Six respondents (1.7%) did not answer enough knowledge items to generate a total knowledge score. For the goal items, the missing responses were also infrequent, with a mean of 2.8% (range 0.4 to 4.4%).

### Reliability

The total knowledge score had an intra-class correlation coefficient (ICC) of 0.75.

The goal items had coefficients ranging from 0.57 to 0.78. The individual coefficients were as follows: “avoid serious risks of chemotherapy such as heart problems and other cancers” ICC = 0.58, “have a treatment that was convenient to complete” ICC = 0.66, “avoid having to take medicine for several years” ICC = 0.67, “avoid the costs of having additional treatments” ICC = 0.69, “live as long as possible” ICC = 0.69, “lower the chance of having cancer come back” ICC = 0.78.

### Validity of knowledge score

The average patient knowledge score, among the 346 (98.3%) patients who completed at least half of the knowledge questions, was 40.1 (SD 20.9). The average provider knowledge score (71.2, SD 17.0) was higher than the patient score (mean difference 31.1, 95% confidence interval 26.9, 35.3). The average healthy volunteer knowledge score (28.8, SD 16.0) was lower than the patient score (mean difference 11.2, 95% confidence interval 5.4, 17.1). Most providers (62%) reported that the knowledge items covered key content extremely or very well. An additional 33% reported that the items covered key content somewhat well. In the written comments, providers did not note any specific missing content.

### Validity of concordance score

These analyses were limited to Stage I patients (n = 232). On multivariable analysis, none of the patient goals was meaningfully associated with receipt of chemotherapy. Age and hormone receptor status were associated with chemotherapy in the multivariable model, with large odds ratios (Table 3). On bivariable analysis, several goals were associated with chemotherapy treatment. For “live as long as possible”, the mean importance score was 9.87 in patients who had chemotherapy and 9.64 in patients who did not (p = 0.008). For “lower the chance of having cancer come back”, the mean importance score was 9.93 in patients who had chemotherapy and 9.77 in those who did not (p = 0.02). For “avoid serious risks of chemotherapy such as heart problems and other cancers”, the mean importance score was 7.01 in patients who had chemotherapy and 7.76 in those who did not (p = 0.076).

**Table 3:** Multivariable logistic regression model of characteristics associated with chemotherapy in Stage I patients

**Characteristic**	**Odds ratio**	**95% CI**
Negative hormone receptor status	14.3	5.51, 37.17
Age (increment of 10)	0.46	0.36 0.59
Live as long as possible	1.45	0.78, 2.66
Have a treatment that was convenient to complete	1.03	1.01, 1.04
Lower the change of having cancer come back	1.78	0.76, 4.17
Avoid the costs of having additional treatments	1.01	0.94, 1.09
Avoid serious risks of chemotherapy	0.93	0.86, 1.01

In the analysis limited to patients with Stage I and hormone receptor positive tumors, no goal was associated with having chemotherapy. Only age was associated with chemotherapy (odds ratio 0.478, 95% confidence interval 0.332, 0.687).

With the multivariable model of treatment, which includes age, hormone receptor status, and goals, 77.6% of patients had treatment that was concordant with the model’s prediction (Table 4). Patients who stated a preference for chemotherapy had higher model-predicted probability of chemotherapy (0.52), compared to those who stated a preference for no chemotherapy or were unsure (0.28, p < 0.0001). Having concordance was not associated with less decisional regret (11.7% for concordant versus 15.4% for non-concordant, p = 0.48) or with more confidence in decisions (8.4 for concordant versus 8.2 for non-concordant, p = 0.67).

**Table 4:** Concordance between predicted and actual treatment for Stage I patients

	**Actual chemotherapy treatment**				**Total**
	**Yes**		**No**		
**Predicted chemotherapy treatment, n (%)**					
Yes, n (%)	43	(18.5)	11	(4.7)	54
No, n (%)	41	(17.7)	137	(59.1)	178
Total*, n (%)	84		148		232

% of the total 232 patients.

*5 patients were not included in the analysis due to missing responses for all goal items.

### Decision process

Patients’ reports about their involvement in the decision process were variable (Table 5). A minority of patients (42.9%) reported that their doctor asked their preference. When asked who made the final decision, 45.7% reported “mainly me”, 22.7% reported “mainly the doctor”, and 29.5% reported “both equally”.

**Table 5:** Decision process by stage

Decision process question*, n (%)	**Stage I**		**Stage II**		**Total**	
Did any doctor tell you that having or not having chemotherapy was a choice for you?						
Yes	169	(71.3)	80	(69.6)	249	(70.7)
No	57	(24.1)	30	(26.1)	87	(24.7)
I am not sure/missing	11	(5.1)	5	(4.3)	16	(4.8)
How much did doctors discuss the reasons to have chemotherapy?						
A lot/some	139	(58.6)	97	(84.3)	236	(67.0)
A little/not at all	80	(33.8)	13	(11.3)	93	(26.4)
I am not sure/missing	18	(7.6)	5	(4.3)	23	(6.5)
How much did doctors discuss reasons not to have chemotherapy?						
A lot/some	119	(50.2)	44	(38.3)	163	(46.3)
A little/not at all	105	(44.3)	61	(53.0)	166	(47.2)
I am not sure/missing	13	(5.5)	10	(8.6)	23	(6.6)
Did any doctor ask you whether you preferred to have chemotherapy?						
Yes	105	(44.3)	46	(40.0)	151	(42.9)
No	103	(43.5)	56	(48.7)	159	(45.2)
I am not sure/missing	29	(12.6)	13	(11.3)	42	(12.2)
Mean decision process score (SD)**	2.2	(1.4)	2.3	(1.2)	2.3	(1.3)

*Questions have been shortened to fit this table.

**Score reflects 1 point for each question answered with yes, a lot, or some (range 0 to 4).

## Discussion

The Breast Cancer Systemic Therapy Decision Quality Instrument is designed to measure the degree to which patients’ chemotherapy decisions are informed and concordant with personal goals. The knowledge score was reliable, and it discriminated among providers, patients, and healthy volunteers. The goal items had modest retest reliability and strong content validity. Patients were able to complete the instrument quickly with few problems.

### Concordance

We were somewhat surprised to find that none of the patients’ goals was meaningfully associated with treatment in the concordance model. Although one goal (“Have a treatment that was convenient to complete”) was statistically significant in the multivariable model, the odds ratio was so close to 1 (1.03) that we did not consider it clinically meaningful. One clinical factor, namely hormone receptor status, was the strongest predictor of receipt of chemotherapy. Hormone receptor status, as well as other clinical and patient features, such as age and tumor stage, should, in fact, guide care decisions. Some clinicians might even argue that providers should recommend chemotherapy to their patients with hormone receptor negative disease. However, for hormone receptor positive patients, it is reasonable for patients’ goals to play a role in determining whether or not to undergo chemotherapy. When we restricted the concordance analyses to stage I, hormone receptor positive participants, we still found that only age was associated with receipt of chemotherapy. None of the goals appeared to have any impact on receipt of chemotherapy, even for hormone receptor positive patients. One interpretation of these results is that providers use clinical features to guide treatment choices, with little regard for patients’ goals and preferences.

Another possible interpretation is that the goal items in this instrument may not have captured aspects that were driving patients’ decisions and as a result, the model may not reflect the tailoring of treatments to patients’ goals. Also, the responses to the goal items had little variability, limiting our ability to discriminate among the treatment options. Although the differences in the mean responses by treatment were statistically significant, the actual magnitude of the differences was quite small and would be difficult to use to guide clinical decisions. Future studies could explore alternative item and response formats for eliciting patients’ goals to increase variability.

A limitation of the concordance analysis is that a few of the Stage I patients may have met guideline criteria for chemotherapy (Her2 neu positive tumor larger than 1 centimeter, triple negative tumor larger than 1 centimeter, or high 21-gene assay score) [1,2]. We did not collect clinical data at that level of detail, however, so we could not remove those patients from the concordance analysis.

### Decision process

Our data on the decision process suggest that providers may not be taking patients’ goals and preferences into account. Less than half of the patients reported that their provider asked about their preferences for chemotherapy. Discussing patients’ goals and preferences is a core element of shared decision making and is important for patient-centered care. Patients and providers may not be viewing chemotherapy as a preference-sensitive decision for stage 1 patients, but instead considering it as effective care. Treatment guidelines, however, clearly state that use of chemotherapy for most patients with stage I disease is dependent upon patients’ preferences [1]. Additional studies may be warranted to understand factors driving providers’ recommendations for use of chemotherapy, including financial and organizational incentives [30].

### Knowledge

The results provide evidence of the reliability and validity of the knowledge score. Few other validated measures of knowledge about chemotherapy are available. A 2003 randomized controlled trial of a decision aid about chemotherapy used a 25-item questionnaire that included true-false items about risk of breast cancer recurrence and risks and benefits of chemotherapy [31]. The questionnaire was based on those used in prior studies of surgery and radiation decisions [32,33], but its psychometric properties have not been reported. Other ad hoc knowledge measures have focused on the accuracy of patients’ estimates of their personal risk of breast cancer recurrence or their estimate of the survival benefit of chemotherapy [17-19,34].

Patient knowledge about key facts related to chemotherapy was quite low in our population, with average overall knowledge of 40.1%. Knowledge about the natural history of early stage breast cancer was particularly low, with only 32.6% of patients answering correctly. Most patients who answered incorrectly overestimated mortality. Knowledge about survival with chemotherapy was low, with 27% of patients answering correctly. Most patients who answered incorrectly overestimated the benefit of chemotherapy on survival.

Prior research suggests that patients tend to overestimate baseline recurrence, mortality rates, and the benefit of chemotherapy; while providers emphasize relative benefit of chemotherapy in terms of impact on 5 or 10 year recurrence [10-12]. This relative benefit is often in the range of 30%, which sounds very high to both patients and providers. When providers educate patients about the absolute benefit of chemotherapy on ten-year mortality (which can range from 2-11%), both parties appear to recognize that the decision is preference-sensitive, and fewer patients elect chemotherapy [9,10,17].

The study had some important limitations. Patients were an average of 2.6 years (SD 9.8) out from treatment, so they may have forgotten some information they knew at the time of decision making. Their responses to questions about goals may have been subject to recall bias, with a tendency to favor goals that were more consistent with the treatment they had. The study population was primarily white, educated, and higher-income than average, so the performance of the instrument in other populations remains unclear. This study was part of a larger study to validate other breast cancer decision quality measures, and the overall response rate for the larger study was lower among non-white patients [35]. We conducted the study at four academic comprehensive cancer centers, where select patients may be referred, and practice styles may differ from that in the community, which may further limit the generalizability of our findings. Healthy volunteers were from one of the sites, so their responses to the knowledge questions may not be representative of healthy persons from other locations.

## Conclusions

The Breast Cancer Systemic Therapy Decision Quality Instrument demonstrated strong reliability and validity as a measure of patient knowledge about chemotherapy for early stage breast cancer in this sample, but its ability to measure concordance was limited. In this population, no patient goal was associated with treatment, and most patients reported they were not asked their preference, suggesting that patients’ goals may not have been adequately considered in treatment decision making about chemotherapy. Further testing will be important to understand the performance of the survey in newly diagnosed patients who are actively making the decision, to evaluate alternative approaches to eliciting patients’ goals, to examine the responsiveness of the knowledge score to interventions, such as patient decision aids, and to gather data to help set benchmarks for knowledge and concordance scores.

## Competing interests

The authors declare that they have no competing interests.

## Authors’ contributions

CL contributed to the study design and participated in the data collection, data interpretation, drafting of the manuscript, and revision of the manuscript. MW participated in the data collection and drafting of the manuscript. YC contributed to the study design and participated in the data management, data analysis, data interpretation, and drafting of the manuscript. JB participated in the data collection, data interpretation, and drafting of the manuscript. BM participated in the data collection, data interpretation, and drafting of the manuscript. AP participated in the data collection, data interpretation, and drafting of the manuscript. KS contributed to the conception and design of the study and participated in the data collection, data interpretation, drafting of the manuscript, and revision of the manuscript. All authors read and approved the final manuscript.

## Pre-publication history

The pre-publication history for this paper can be accessed here:

http://www.biomedcentral.com/1472-6947/14/73/prepub

## Supplementary Material

Additional file 1 Breast Cancer Systemic Therapy Decision Quality Instrument. Click here for file
