# Supplementary material for: Measuring decision quality: psychometric evaluation of a new instrument for breast cancer chemotherapy
Source: BMC Med Inform Decis Mak. 2014 Aug 20;14:73. doi: 10.1186/1472-6947-14-73 (PMC4150558; doi:10.1186/1472-6947-14-73)
Supplement: Additional file 1 — Breast Cancer Systemic Therapy Decision Quality Instrument. [file 1472-6947-14-73-S1.doc]

**Additional file 1. Breast Cancer Systemic Therapy Decision Quality Instrument**

Note that the instrument includes questions about hormone therapy, which are not discussed in this manuscript.

**SECTION 3: TREATMENTS AFTER SURGERY**

**CHEMOTHERAPY AND HORMONE THERAPY**

**Please answer these questions about your experiences talking with doctors, nurses and other health care professionals about whether or not to have more treatment after surgery. The treatments in this section are medicines given after surgery, chemotherapy and hormone therapy (such as tamoxifen or Arimidex).**

3.1. Did any of your doctors tell you that having or not having chemotherapy was a choice for you?

🞏 Yes

🞏 No

🞏 I am not sure

3.2. Did any of your doctors tell you that having or not having hormone therapy was a choice for you?

🞏 Yes

🞏 No

🞏 I am not sure

3.3. Did any of your doctors express an opinion or make a recommendation about whether you should have chemotherapy and hormone therapy?

🞏 Yes

🞏 No **🡪 If no, GO TO 3.5**

3.4. What did the doctors think you should have?

🞏 Chemotherapy only

🞏 Hormone therapy only

🞏 Both chemotherapy and hormone

therapy

🞏 Not have chemotherapy and not

have hormone therapy

🞏 Different doctors made different

recommendations

3.5. Who made the final decision about these treatments, mainly the doctor, mainly you, or both you and your doctor, equally?

🞏 Mainly the doctor

🞏 Mainly me

🞏 Both equally

3.6. How much were you involved in making the decision about these treatments for breast cancer?

🞏 More than I wanted

🞏 About as much as I wanted

🞏 Less than I wanted

3.7. How much did doctors discuss the reasons to have chemotherapy with you?

🞏 A lot

🞏 Some

🞏 A little

🞏 Not at all

🞏 I am not sure

3.8. How much did doctors discuss the reasons not to have chemotherapy with you?

🞏 A lot

🞏 Some

🞏 A little

🞏 Not at all

🞏 I am not sure

3.9. How much did doctors discuss the reasons to have hormone therapy with you?

🞏 A lot

🞏 Some

🞏 A little

🞏 Not at all

🞏 I am not sure

3.10. How much did doctors discuss the reasons not to have hormone therapy with you?

🞏 A lot

🞏 Some

🞏 A little

🞏 Not at all

🞏 I am not sure

3.11. Did any of your doctors ask you whether you preferred to have chemotherapy, hormone therapy, both or neither?

🞏 Yes

🞏 No

🞏 I am not sure

3.12. Which treatment was your personal

preference?

🞏 Chemotherapy only

🞏 Hormone therapy only

🞏 Both chemotherapy and hormone

therapy

🞏 Not have chemotherapy and not

have hormone therapy

🞏 I am not sure

3.13. Did you have chemotherapy?

🞏 Yes

🞏 No **🡪 If no, GO TO 3.15**

3.14. What was the main reason that you had chemotherapy?

🞏 Doctor said it was my only option

🞏 Doctor recommended it

🞏 My personal preference

🞏 Family or friends recommended it

🞏 Other reason:________________

3.15. Did you have hormone therapy?

🞏 Yes

🞏 No **🡪 If no, GO TO 3.17**

3.16. What was the main reason that you had

hormone therapy?

🞏 Doctor said it was my only option

🞏 Doctor recommended it

🞏 My personal preference

🞏 Family or friends recommended it

🞏 Other reason:________________

3.17. On a scale from 0 to 10, where 10 means extremely well informed and 0 means not informed at all, how informed did you feel about chemotherapy treatments?

🞏 10 Extremely well informed

🞏 9

🞏 8

🞏 7

🞏 6

🞏 5 Somewhat informed

🞏 4

🞏 3

🞏 2

🞏 1

🞏 0 Not informed at all

3.18. On a scale from 0 to 10, where 10 means extremely well informed and 0 means not informed at all, how informed did you feel about hormone therapy treatments?

🞏 10 Extremely well informed

🞏 9

🞏 8

🞏 7

🞏 6

🞏 5 Somewhat informed

🞏 4

🞏 3

🞏 2

🞏 1

🞏 0 Not informed at all

3.19. On a scale from 0 to 10, where 10 means

extremely confident and 0 means not at all

confident, how confident were you that the

decision about chemotherapy was the right one?

🞏 10 Extremely confident

🞏 9

🞏 8

🞏 7

🞏 6

🞏 5 Somewhat confident

🞏 4

🞏 3

🞏 2

🞏 1

🞏 0 Not confident at all

3.20. On a scale from 0 to 10, where 10 means extremely confident and 0 means not at all confident, how confident were you that the decision about hormone therapy was the right one?

🞏 10 Extremely confident

🞏 9

🞏 8

🞏 7

🞏 6

🞏 5 Somewhat confident

🞏 4

🞏 3

🞏 2

🞏 1

🞏 0 Not confident at all

3.21. If you had to do it again, would you

have the same treatments?

🞏 Definitely have the same

🞏 Probably have the same

🞏 Not sure

🞏 Probably not have the same

🞏 Definitely not have the same

**Please go on to the next page 🡪**

**The next set of questions includes some reasons other women with breast cancer have given for choosing chemotherapy or hormone therapy. Please mark on a scale from 0 to 10, where 10 is extremely important and 0 is not at all important, how important each of the following were for your decision about whether or not to have these treatments.**

3.22. How important was it to you to live as long as possible?

🞏 10 Extremely Important to me

🞏 9

🞏 8

🞏 7

🞏 6

🞏 5 Somewhat important to me

🞏 4

🞏 3

🞏 2

🞏 1

🞏 0 Not at all important to me

3.23. How important was it to you to have a treatment that was convenient to complete?

🞏 10 Extremely Important to me

🞏 9

🞏 8

🞏 7

🞏 6

🞏 5 Somewhat important to me

🞏 4

🞏 3

🞏 2

🞏 1

🞏 0 Not at all important to me

3.24. How important was it to you to lower the chance of having cancer come back?

🞏 10 Extremely Important to me

🞏 9

🞏 8

🞏 7

🞏 6

🞏 5 Somewhat important to me

🞏 4

🞏 3

🞏 2

🞏 1

🞏 0 Not at all important to me

3.25. How important was it to you to avoid the costs of having additional treatments?

🞏 10 Extremely Important to me

🞏 9

🞏 8

🞏 7

🞏 6

🞏 5 Somewhat important to me

🞏 4

🞏 3

🞏 2

🞏 1

🞏 0 Not at all important to me

3.26. How important was it to you to avoid serious risks of chemotherapy such as heart problems and other cancers?

🞏 10 Extremely Important to me

🞏 9

🞏 8

🞏 7

🞏 6

🞏 5 Somewhat important to me

🞏 4

🞏 3

🞏 2

🞏 1

🞏 0 Not at all important to me

3.27. Did any of your doctors tell you that hormone therapy was an option for you?

🞏 Yes, hormone therapy was an

option for me

🞏 No, hormone therapy was not an

option for me **🡪 If no, GO TO 3.30**

3.28. How important was it to you to avoid serious risks of hormone therapy such as other cancers or bone problems?

🞏 10 Extremely Important to me

🞏 9

🞏 8

🞏 7

🞏 6

🞏 5 Somewhat important to me

🞏 4

🞏 3

🞏 2

🞏 1

🞏 0 Not at all important to me

3.29. How important was it to you to avoid having to take medicine for several years?

🞏 10 Extremely Important to me

🞏 9

🞏 8

🞏 7

🞏 6

🞏 5 Somewhat important to me

🞏 4

🞏 3

🞏 2

🞏 1

🞏 0 Not at all important to me

3.30. Of the reasons you just rated, which do you think are the three most important for your decision about whether or not to have chemotherapy or hormone therapy for breast cancer? Please circle the letter next to the three most important reasons for you.

A. Live as long as possible

B. Lower chance of cancer coming back

C. Convenient to do

D. Avoid costs of additional treatments

E. Avoid serious risks of chemotherapy

F. Avoid serious risks of hormone therapy

G. Avoid taking medicine for several years

**Please go on to next page 🡪**

**The next set of questions asks about the breast cancer treatments chemotherapy and hormone therapy. The correct answer to each question is based on medical research and practice. Please do your best to answer these questions using what you know now, even if you did not have one or more of the treatments discussed in the questions.**

3.31. For most women with early breast cancer,

how much would waiting 4 weeks to make a treatment decision about chemotherapy and hormone therapy affect their chances of survival?

🞏 A lot

🞏 Somewhat

🞏 A little or not at all

🞏 I am not sure

3.32. For each of the following, please mark whether or not it increases the chance of having breast cancer come back anywhere in the body?

| a. | Having high blood pressure | 🞏 Yes | 🞏 No | 🞏 Not  sure |
| --- | --- | --- | --- | --- |
| b. | Being older than 50 ………..…….. | 🞏 Yes | 🞏 No | 🞏 Not  sure |
| c. | Having a large tumor …………. | 🞏 Yes | 🞏 No | 🞏 Not  sure |
| d. | Having cancer in lymph nodes.….. | 🞏 Yes | 🞏 No | 🞏 Not  sure |

3.33. Without chemotherapy or hormone therapy, about how many women with early stage breast cancer will eventually die of breast cancer?

🞏 More than half

🞏 About half

🞏 Less than half

🞏 I am not sure

3.34. Without chemotherapy or hormone therapy, about how many women with early stage breast cancer will be cancer free in ten years?

🞏 More than half

🞏 About half

🞏 Less than half

🞏 I am not sure

3.35. How long does it usually take to complete chemotherapy treatment for breast cancer?

🞏 Less than 1 month

🞏 1 to 6 months

🞏 7 months to 1 year

🞏 More than 1 year

🞏 I am not sure

3.36. Can breast cancer come back after chemotherapy?

🞏 Yes

🞏 No

🞏 I am not sure

3.37. Out of 100 women who have chemotherapy for breast cancer, how many will have a serious side effect, such as getting another kind of cancer or serious heart problem?

🞏 Fewer than 5

🞏 5-10

🞏 11-20

🞏 More than 20

🞏 I am not sure

3.38. For each of the following, mark whether or not some women have this side effect from chemotherapy.

| a. | Hair loss……… | 🞏 Yes | 🞏 No | 🞏 Not  sure |
| --- | --- | --- | --- | --- |
| b. | Sexual problems… | 🞏 Yes | 🞏 No | 🞏 Not  sure |
| c. | Breast pain……. | 🞏 Yes | 🞏 No | 🞏 Not  sure |
| d. | Not able to have children (infertility) | 🞏 Yes | 🞏 No | 🞏 Not  sure |

3.39. On what kind of tumors does hormone therapy work best?

🞏 Hormone receptor positive tumors

🞏 Hormone receptor negative tumors

🞏 It works the same on both kinds of tumors

🞏 I am not sure

3.40. Can breast cancer come back after hormone therapy?

🞏 Yes

🞏 No

🞏 I am not sure

3.41. As treatment for early stage breast cancer, how often is hormone therapy usually given?

🞏 Once a week for several years

🞏 Every day for several years

🞏 One time only

🞏 I am not sure

3.42. Out of 100 women who have hormone therapy for breast cancer, how many have a serious side effect, such as getting another kind of cancer or life-threatening blood clot?

🞏 Fewer than 5

🞏 5-10

🞏 11-20

🞏 More than 20

🞏 I am not sure

3.43. For each of the following, mark whether or not some women have this side effect from hormone therapy.

| a. | Hair loss……….... | 🞏 Yes | 🞏 No | 🞏 Not  sure |
| --- | --- | --- | --- | --- |
| b. | Sexual problems…. | 🞏 Yes | 🞏 No | 🞏 Not  sure |
| c. | Breast pain………. | 🞏 Yes | 🞏 No | 🞏 Not  sure |
| d. | Not able to have children (infertility)… | 🞏 Yes | 🞏 No | 🞏 Not  sure |

**For the next four questions, you may not know the exact number, but please take your best guess.**

3.44. If 100 women with breast cancer like yours had chemotherapy, about how many would be cancer free in ten years?

_________ write in number of people

🞏 I am not sure

3.45. If 100 women with breast cancer like yours had hormone therapy, about how many would be cancer free in ten years?

_________ write in number of people

🞏 I am not sure

3.46. If 100 women with breast cancer like yours had **both** chemotherapy and hormone therapy, about how many would be cancer free in ten years?

_________ write in number of people

🞏 I am not sure

3.46 If 100 women with breast cancer like yours did **not** have chemotherapy and did **not** have hormone therapy, about how many would be cancer free in ten years?

_________ write in number of people

- I am not sure

3.47. For each of the following, please mark whether or not it was true for you when you were making a choice about chemotherapy for breast cancer.

| a. | Did you feel that you knew the benefits and risks of each option? | 🞏 Yes | 🞏 No | 🞏 Not  sure |
| --- | --- | --- | --- | --- |
| b. | Were you clear about which benefits and risks mattered most to you? | 🞏 Yes | 🞏 No | 🞏 Not  sure |
| c. | Did you have enough support and advice to make a choice? | 🞏 Yes | 🞏 No | 🞏 Not  sure |
| d. | Did you feel sure about the best choice for you? | 🞏 Yes | 🞏 No | 🞏 Not  sure |

3.48. Are there any other comments about the survey or your experiences selecting treatments for breast cancer that you would like to share? (Please write in):

______________________________________________________________________________________________________________________________________________________________________________________________________

**Glossary**

**Breast conserving surgery**: It is also called Lumpectomy. The surgeon makes an incision in the breast and removes the tumor along with a border of healthy tissue all around it.

**Breast reconstruction**: It is surgery to re-create a breast shape using a breast implant, tissue from another part of your body (called flaps), or a combination.

**Chemotherapy**: Medications that can kill cancer cells throughout the body. Some are taken by mouth, and others are given by needle/IV.

**DCIS**: Ductal carcinoma in situ. An early form of breast cancer (called Stage 0) where the cancer cells are contained in the lining of a breast duct.

**Flaps**: Using skin and tissue from other parts of the body (usually the stomach or back) to re-create the breast shape.

**Hormone receptor**: A protein on the surface of a cell that binds to a specific hormone. Breast cancer cells that have estrogen and/or progesterone receptors and are called hormone receptor positive.

**Hormone therapy**: Medication that blocks or removes hormones that many breast cancer tumors need to grow. Types of medications include tamoxifen, anastrozole (Arimidex®), exemestane (Aromasin®), and letrozole (Femara®). May also be called endocrine therapy or anti-hormonal therapy.

**Implants**: A fluid filled sac that is used to re-create a breast shape.

**Lumpectomy**: (also called breast conserving surgery) is surgery to remove only the breast tumor and a border of healthy tissue around it, saving the breast.

**Lymph nodes**: Small clusters of tissues that help defend the body from the spread of infections and cancer. The lymph nodes under the armpit are often checked to see whether breast cancer has spread.

**Mastectomy**: Surgery to remove the entire breast.

**Radiation therapy**: The use of high-energy x-rays to kill cancer cells. Radiation is a local therapy used to kill cancer cells that may remain in the breast area after surgery.

**Sentinel node biopsy**: The surgeon injects dye and uses it to identify and remove the sentinel lymph node(s) under the armpit and checks for the presence of cancer cells.

**Tamoxifen**: A type of hormone therapy medication.
